# At the crossroads of biomacromolecular research: highlighting the interdisciplinary nature of the field

**DOI:** 10.1186/1752-153X-1-4

**Published:** 2007-02-19

**Authors:** Dennis R Livesay

**Affiliations:** 1 Department of Computer Science and Bioinformatics Research Center, University of North Carolina at Charlotte, Charlotte, NC 28223 USA.

## Abstract

Due to their complexity and wide-ranging utility, biomacromolecular research is an especially interdisciplinary branch of chemistry. It is my goal that the Biomacromolecules subject area of Chemistry Central Journal will parallel this richness and diversity. In this inaugural commentary, I attempt to set the stage for achieving this by highlighting several areas where biomacromolecular research overlaps more traditional chemistry sub-disciplines. Specifically, it is discussed how Materials Science and Biotechnology, Analytical Chemistry, Cell Biology and Chemical Theory are each integral to modern biomacromolecular research. Investigators with reports in any of these areas, or any other dealing with biomacromolecules, are encouraged to submit their research papers to Chemistry Central Journal.

## Background

Chemistry, often referred to as the *central science*, is critical to a fundamental understanding of the world around us. Chemical concepts have traditionally been central to the canonical sciences (i.e., biology, physics, and geology). As a chemist, it is gratifying to see the importance of chemistry continue in newer disciplines as well (i.e., materials science, forensics, astrobiology, biotechnology, bioinformatics, pharmacology, and atmospheric science). *Chemistry Central Journal *ambitiously aims to cover this rich diversity within modern chemical research. A quick perusal of the over fifty different subsections included within the journal reinforces this point. Moreover, many of these subsections are varied and interdisciplinary in their own right. This is especially true when considering biomacromolecular research.

Current biomacromolecular research is synonymous with physics, biology, and chemistry - clearly, a proper understanding of the physiochemical properties of these large molecules and how they function within the cell requires molecular-level insight. From a more practical point-of-view, figuring out ways to harness their plasticity is being vigorously pursued within electrical and nanoengineering, polymer science, and biotechnology. In order to parallel this breadth, the biomacromolecules section will be as inclusive as possible by featuring reports with both fundamental science and more applied points of view. For example, a sampling of the research topics that are appropriate includes:

• Bioenergetics

• Biomacromolecular function

• Biomaterials and biocatalysts

• Biomedical applications (biosensors, drug delivery devices, etc.)

• Biomimicry and molecular design

• Crowding and other *in vivo *effects

• Folding (experiment and theory)

• Molecular recognition and biomacromolecular assembly

• Physical chemistry of biological macromolecules

• Proteomics

• Single molecule studies

• Structural biology

In this inaugural commentary, I try to set the stage for this Gestalt view of biomacromolecular research by briefly highlighting examples of important issues in the overlap between biomacromolecules and several other topical research areas. In each of the exemplar discussions, key aspects of biomacromolecular research occur at the intersection of multiple scientific paradigms. While it is impossible to identify all research avenues that overlap with biomacromolecules in such a short commentary, hopefully a common interdisciplinary *spirit *is conveyed.

## Materials Science and Biotechnology

The overlap with materials and polymers is one of the most important research areas within biomacromolecules. The primary aim of which is to understand and manipulate materials at a fundamental level. Similarly, self-assembly and related nanotechnology efforts have a keen interest in biomaterials due to their exquisite specificity and efficiency. The Holy Grail is to develop medical implants that look, behave, and function like the biological systems they replace. It follows that one of the most important areas of biomaterials research attempts to figure out ways to escape a devastating immune response [1]. Moreover, understanding how these foreign biomaterials interact with tissues and/or the biological milieu is equally important. However, the utility of biomaterials is not limited to just biological problems. For example, it has recently been demonstrated, with much fanfare, that the electronic properties of DNA can be exploited to construct electrical circuits. This exciting observation introduces a whole new framework in which to develop smaller and more specific electronic devices [2]. A third important biomacromolecular research topic is biomimicry, which attempts to develop *bio-inspired *concepts. The guiding principle behind biomimicry is that evolution has resulted in highly optimized structures that can be transferred to modern technologies.

Likewise, the field of biotechnology also aims to strengthen the overlap between engineering principles and biological efforts. Biomacromolecules are commonly studied within biotechnology research due to their potential as drug-delivery devices, artificial tissues, scaffolding for artificial organs, etc. Encapsulation of therapeutic molecules within synthetic polymers provides a means to escape toxic side effects, control release rate, and target drug delivery. The importance of artificial tissues and organs is self-evident; however, the technical challenges posed by these tasks remain daunting. On a much smaller scale, *de novo *design and intelligent redesign of enzymes to catalyze specific industrial and environmentally related (remediation) reactions is being enthusiastically pursued due to the unparalleled specificity imparted by enzyme catalysts [3]. Unfortunately, the expectancy of all these efforts continues to surpass our current capacity.

## Analytical Chemistry

Shifting to a more fundamental point of view, structural studies form the foundation of nearly all biomacromolecular research. Linus Pauling, Jim Watson, Francis Crick, John Kendrew, Max Perutz, and many others revolutionized our understanding of proteins and nucleic acids [4]. Their pioneering work paved the way for today's more applied efforts. This structural context has helped set paradigms in modern biology about the origins of life, including: molecular genetics, enzymology, and energy transduction. In the decades since the genesis of structural biology, the Protein Data Bank has been primarily filled up with large numbers of globular proteins. This conspicuous enrichment of globular proteins has occurred because scientists are pragmatic; globular proteins represent the "low hanging fruit." The next challenge is to characterize portions of the proteome that are less amendable to current structural techniques. The recent explosion in the number of structural studies related to membrane-bound proteins has provided key insight into this segment of the proteome. Roderick McKinnon's groundbreaking work on potassium channels illuminates the molecular basis of electrophysiology, provides a framework for designing new therapeutics, and (perhaps most importantly) confirms that all-atom structural biology of membrane-bound proteins is feasible [5].

The next pieces of the proteome likely to generate many breakthroughs are: (1) natively disordered proteins that have no structure as traditionally defined, and (2) massive macromolecular complexes. As their name implies, natively disordered proteins (aka, intrinsically unfolded proteins) challenge our very concept of *structural *biology [6]. These proteins compose 30-40% of the proteome, and appear to be commonly associated with cell signaling and/or responses to environmental stresses. Elucidating their un-structured characteristics, or the extent thereof, their expression profiles, and identifying the molecular counterparts they interact with should yield unfathomable insight. On a different front, large biological assemblies present many technical challenges to current analytical techniques. The lengthscales of these complexes prohibit characterization by standard methods (primarily x-ray crystallography and NMR). A sensible approach is to divide-and-conquer, where structural characterization of constituent subunits using standard techniques is followed by reassembly [7]. Reassembly requires a synergism of measurement and theory - computational modeling informed by basic connections from experiment should provide realistic models of the complexes. The most promising experimental techniques to elucidate the connectivity of these assemblies include: footprinting, cross-linking + mass spectrometry, and imaging methods. Once we have these tools in hand, we will be better equipped to investigate the cell's remarkable molecular machines that escape our current capabilities.

## Cell Biology

With the exception of structural studies, most of our current understanding of the physical properties of biomacromolecules comes from *in vitro *experiments within dilute solutions. This reductionist viewpoint has been necessary in order to make experimental interpretation tractable. However, this is clearly a drastic simplification; the cell is not a dilute solution! The consequences of this are profound. First, the cytoplasm of a cell is more gel-like than liquid. Second, the water molecule concentration of the cytoplasm is significantly reduced from bulk due to the presence of ions, small molecules, proteins, and organelles. And third, the local water molecule density is variable throughout the cell. All three of these observations can have pronounced effects on macromolecular structure, function, and stability. One of the most hotly researched topics in this area concerns the effects of molecular crowding. The congested cellular milieu significantly affects the physiochemical properties of its constituent molecules. As previously expected, and now confirmed, an important consequence of crowding is that the equilibrium between folded and unfolded states is shifted towards native-like populations [8]. The explanation for this is based on straightforward size considerations; the volume occupied by an unfolded protein is significantly greater, and thus less likely within a crowded environment, than its native-like counterpart. A better appreciation for these types of environmental effects will get us closer to understanding the underlying physics and chemistry of life. Moreover, it will move us ever close to designed biomacromolecular devices that behave and perform like naturally evolved functionalities.

## Chemical Theory

The first step towards any rational molecular design effort is to understand the underlying physics and chemistry of the system in question. Regrettably, application of standard (small molecule) chemical theory paradigms to macromolecules routinely fails to reproduce experimentally observable metrics. This performance gap is due to the extraordinary complexity of these large and highly interconnected polymers. For example, additivity of constituent free energy components is a common theme in small molecule chemistry; however, nonadditivity is frequently observed within protein double-mutant cycles [9]. Nevertheless, free energy nonadditivity is rarely considered in coarse-grained energy functions used to speed up theoretical investigations. In order for chemical theory to better recapitulate experimental observations, this problem must be tackled. A second problem arises from timescales [10]. The timescales associated with biological processes can span over fifteen orders of magnitude. Stated slightly differently, they overlap at least three different levels of modern chemical theory (quantum, molecular, and Brownian). The 1990's saw a large number of Federally funded research projects address this multi-timescale problem; unfortunately, a solution remains as elusive as ever. A third problem arises from the simple fact that biopolymers are molecules. This statement is not meant to be trite, but to draw attention to the fact that (like all molecules) biological macromolecules have intramolecular motions. In fact, due to size, these intramolecular motions can be amplified into drastic conformational changes. The most obvious way to theoretically examine intramolecular motions is via molecular dynamics simulations. Unfortunately, their utility is limited due to immense computational cost; this is especially true when considering large macromolecular complexes. Several elastic-network models [11] and graph-theoretic mechanical models [12] of flexibility have become popular recently due to their efficiency; however, these methods know nothing about thermodynamics and temperature. A key challenge of biomacromolecular theory is to develop methods that bridge the mechanical models with thermodynamics [13]. High quality reports that tackle fundamental problems associated with biomacromolecular theory are especially encouraged.

## Conclusion

Due to the innate complexity of proteins, nucleic acids, and polysaccharides, a proper understanding of biological macromolecules requires a diverse array of experimental and theoretical techniques. Moreover, as highlighted above, biomacromolecular research overlaps many different fields ranging from biomedicine to engineering. It should further be pointed out that it is impossible to completely segregate these interdisciplinary connections. For example, biomacromolecules as sensors of normal and pathological cellular function overlap all four areas discussed above. Theoretical methods provide the foundation upon which such sensors can be devised. The realization of such methods, which can are commonly based on FRET, fluorescence, electrochemical gradients, etc., is part and parcel to analytical chemistry. Developing the sensors into commercially viable devices is in-line with materials and engineering. Finally, interpretation of and reaction to the *in vivo *readings is emerging as a cornerstone of cellular biology. With all of this in mind, it is my goal to keep the biomacromolecules section as diverse as the *Chemistry Central Journal *itself. Above, I highlighted a few of the most confounding research tasks related to biomacromolecules currently receiving great attention amongst a myriad number of research focus areas. All aspects about biomacromolecules are appropriate and welcome for submission. If you have any questions about submitting a biomacromolecules research report, please feel free to contact me.
